# Structural plasticity of mouse intestinal microvilli revealed by tissue-level cryo-ET

**DOI:** 10.52601/bpr.2025.250038

**Published:** 2026-08-31

**Authors:** Jiaming Liu, Xiaoyu Tang, Peng Wang, Huanhuan Huang, Zihang Yu, Jingtao Zheng, Ying Liu, Meijing Li

**Affiliations:** 1 Institute of Bio-Architecture and Bio-Interactions, Shenzhen Medical Academy of Research and Translation, Shenzhen 518107, Guangdong, China

**Keywords:** Cryo-electron tomography (cryo-ET), Cryo-focused ion beam (cryo-FIB), Brush border, Branched microvilli, Actin cytoskeleton

## Abstract

Cryo-electron tomography (cryo-ET) enables three-dimensional imaging of cellular architecture in a near-native state, but its use in intact mammalian tissues remains technically challenging. Here, using an optimized tissue-level cryo-ET pipeline that integrates high-pressure freezing, cryo-CLEM, and an improved serial lift-out cryo-FIB workflow, we reconstructed brush-border regions of mouse small intestine from 43 tomographic tilt series and analyzed 490 individual microvilli. Microvilli formed a quasi-regular lattice (mean inter-microvillar spacing ≈ 61 nm) with diameters of 72−114 nm and clearly resolved actin core bundles. Each protrusion bore lateral, nanobristle-like projections that were morphologically heterogeneous and, in rare cases, bridged adjacent microvilli. Importantly, we identified 27 branched microvilli (~5.5%), including Y-shaped and multi-branched forms; the organization of actin bundles in these structures is consistent with a tip-to-base division mechanism for generating daughter protrusions. These observations reveal previously underappreciated structural plasticity in the mammalian brush border and support a fission-like pathway for microvillus renewal.

## INTRODUCTION

Microvilli are actin-rich, membrane-bound protrusions that extend from the apical surface of intestinal epithelial cells, significantly increasing the surface area available for nutrient absorption while also contributing to barrier integrity and sensory functions (Sauvanet *et al.* 2015). Each microvillus is supported by a core of approximately 20−30 parallel actin filaments, tightly bundled and stabilized by crosslinking proteins such as villin, fimbrin, and espin (Meenderink *et al.* 2019). In absorptive epithelia of the intestine, kidney, and other organs, microvilli are organized into dense, highly ordered arrays known as brush borders, which adopt a hexagonal lattice architecture. These specialized structures are critical for normal physiological function; disruptions in microvillar organization are associated with malabsorption, sensory defects, and inflammatory pathology (Schneeberger *et al.* 2018; Shifrin and Tyska 2012).

A single enterocyte may harbor thousands of microvilli, thereby maximizing luminal interface and absorptive capacity (Mooseker 1985). Given the continuous renewal of the intestinal epithelium, microvilli undergo constant assembly and turnover throughout life (Sauvanet *et al.* 2015). Notably, mature microvilli display remarkable uniformity in both size and spacing, suggesting a tightly regulated biogenesis process. Key molecular determinants include protocadherin-based intermicrovillar adhesion complex (IMAC) and associated scaffolding proteins, which mediate alignment and packing of adjacent microvillar tips (Crawley *et al.* 2014; Meenderink *et al.* 2019). Despite this understanding, fundamental questions persist regarding microvillar plasticity, particularly the mechanisms underlying the generation of new microvilli and the remodeling of existing ones during physiological turnover.

Historically, branched "Y-shaped" microvilli have been observed in regenerating intestinal epithelium, raising the hypothesis that microvilli may arise through a fission-like mechanism (Tilney and Cardell 1970). Our previous *in vivo* cryo-ET studies in *C. elegans* have provided structural evidence supporting this model, revealing branched microvilli with actin core configurations consistent with tip-to-base fission (Zhu *et al.* 2022). Moreover, disruption of lateral adhesive structures − nanoscale projections termed "nanobristles" − results in increased prevalence of Y-shaped microvilli, suggesting that these morphological intermediates are functionally linked to renewal processes (Zhu *et al.* 2022). Live imaging study also observed that new microvilli emerge from pre-existing protrusions (Gaeta *et al.* 2021). These findings point to a role for actin-driven branching in brush border regeneration; however, the extent to which such mechanisms operate in mammalian systems has remained uncertain.

To address these gaps, we optimized a tissue-level cryo-electron tomography pipeline for the mouse small intestine to visualize microvilli *in situ* at nanometer resolution. This approach preserves native ultrastructure and enables three-dimensional analysis of both membrane and cytoskeletal organization. Our tomographic reconstructions reveal canonical actin-cored microvilli decorated with lateral filamentous projections and a previously underappreciated diversity of branched morphologies, including bifurcated and multi-branched forms. Importantly, the arrangement of actin bundles within branched structures is consistent with a tip-to-base division model, and the properties of mammalian nanobristles differ from those reported in nematodes, suggesting species-specific structural and mechanical features.

Here, we present these *in situ* structural observations and discuss their implications for brush-border renewal and mechanical organization. By providing direct three-dimensional evidence of branched microvilli and heterogeneous lateral projections in the mammalian intestine, our study broadens the conceptual framework for how epithelial surfaces maintain and remodel their absorptive architecture and highlights specific hypotheses for the molecular and physiological regulation of microvillar branching.

## RESULTS

### Optimized tissue-level cryo-ET workflow for visualizing mouse intestinal microvilli

**Figure 1:**
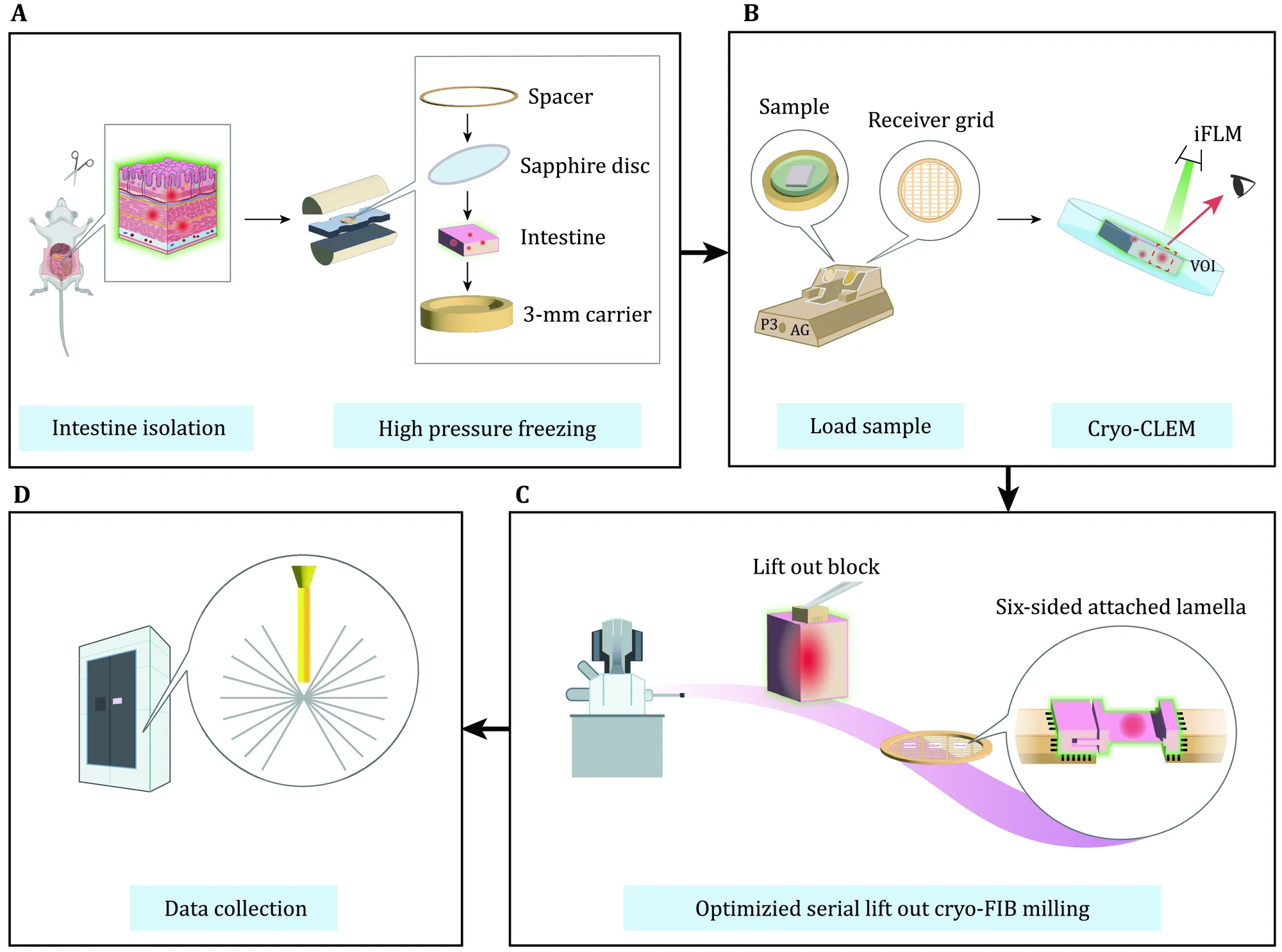
Overview of the optimized cryo-ET workflow for mouse intestine sample preparation. **A** The mouse intestine was isolated and rapidly frozen using a modified HPF protocol. **B** Vitreous intestine samples were transferred to a Hydra plasma-FIB system, and the volume of interest (VOI) was localized using cryo-CLEM. **C** A cryo-intestinal lamella was prepared using the optimized serial lift-out cryo-FIB milling protocol. **D** Tomographic tilt series were acquired using cryo-TEM

**Figure 2:**
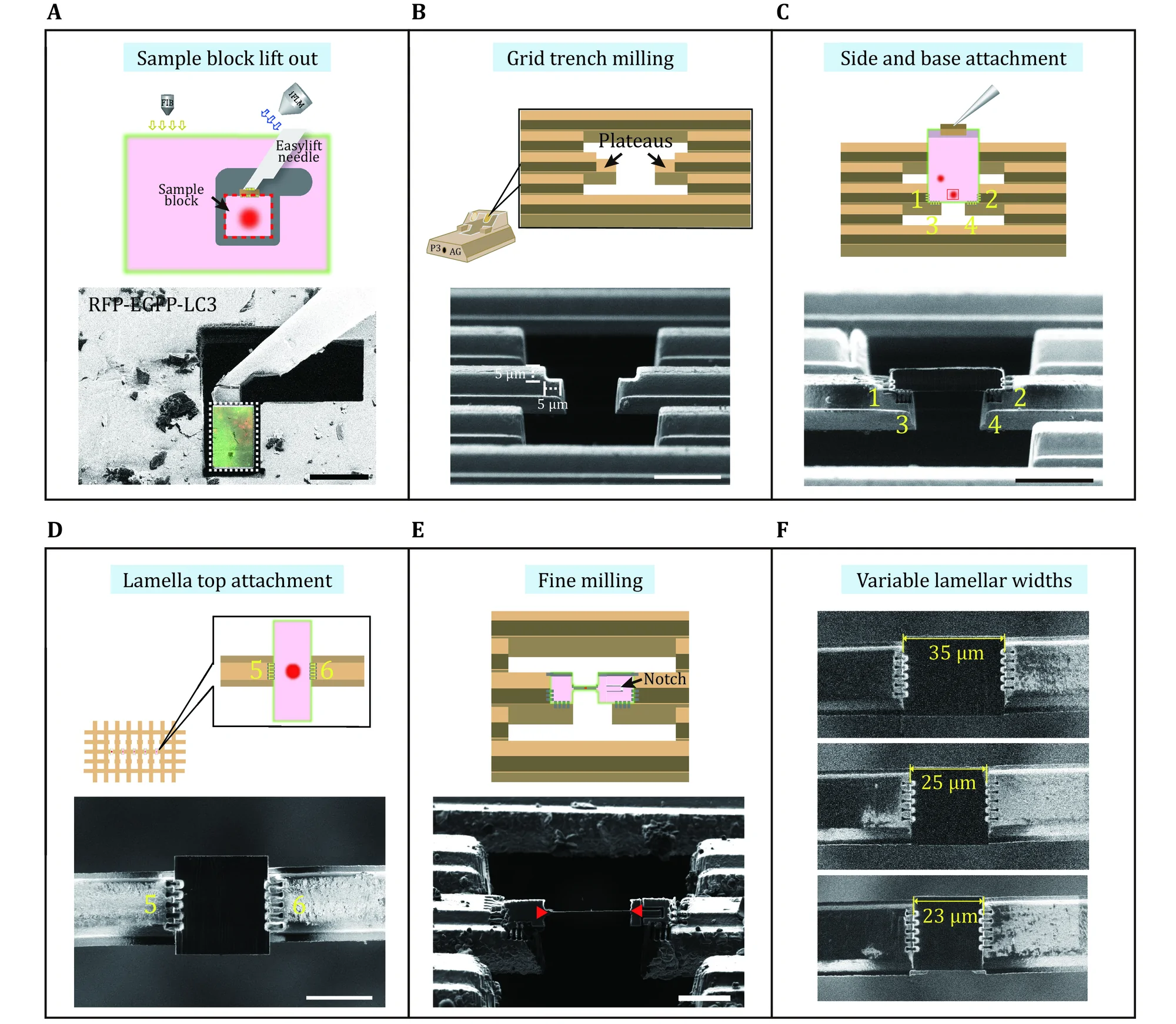
Optimized Serial Lift Out cryo-FIB milling workflow. **A** A sample block, indicated by a dashed rectangle at the VOI position showing fluorescent signals, was milled out from the entire sample block and lifted out using the EasyLift nanomanipulator; scale bar = 50 μm. **B** Trench milling was conducted on the receiver grid to create two plateaus for lamella attachment. The width and height of each plateau were 5 μm and 5 μm, respectively; scale bar = 20 μm. **C** A first lamella was generated by attaching it to both sides (indicated by 1 and 2) and the bottom (indicated by 3 and 4) of the two plateaus. The remaining sample block was then released using a line pattern milling strategy; scale bar = 20 μm. **D** The lamella was further secured on both sides of the top surfaces (indicated by 5 and 6) of the two plateaus; scale bar = 20 μm. **E** Fine milling was performed to polish the lamella into a thin section with a thickness of approximately 200 nm; scale bar = 10 μm. **F** Three lamellae of varying widths prepared using the optimized workflow

To investigate the microvillar architecture of the mouse intestine, we developed an optimized, tissue-level cryo-ET pipeline that integrates high-pressure freezing (HPF) (Fig. 1A), cryo-correlative light and electron microscopy (cryo-CLEM) (Fig. 1B), and an improved serial lift-out cryo-focused ion beam (cryo-FIB) milling strategy (Fig. 1C; supplementary Movie S1; see also step-by-step protocol (Tang *et al*. 2025)), followed by tilt series acquisition (Fig. 1D). Fluorescent signals from RFP-EGFP-LC3 transgenic mice facilitate precise localization of intestinal villi (Fig. 2A). Subsequent serial lift-out cryo-FIB milling enables the fabrication of tissue lamellae from targeted intestinal villi with variable orientations (Fig. 2; supplementary Movie S1). Our six-sided attachment approach provides enhanced mechanical stability and reduces dependence on operator expertise compared to conventional serial lift-out procedures (Schiøtz *et al.* 2024). The optimized protocol consistently yields vitrified lamellae of sufficient quality for high-resolution cryo-ET imaging, ensuring excellent preservation of ultrastructural integrity.

### Organization of mouse intestinal microvilli

**Figure 3:**
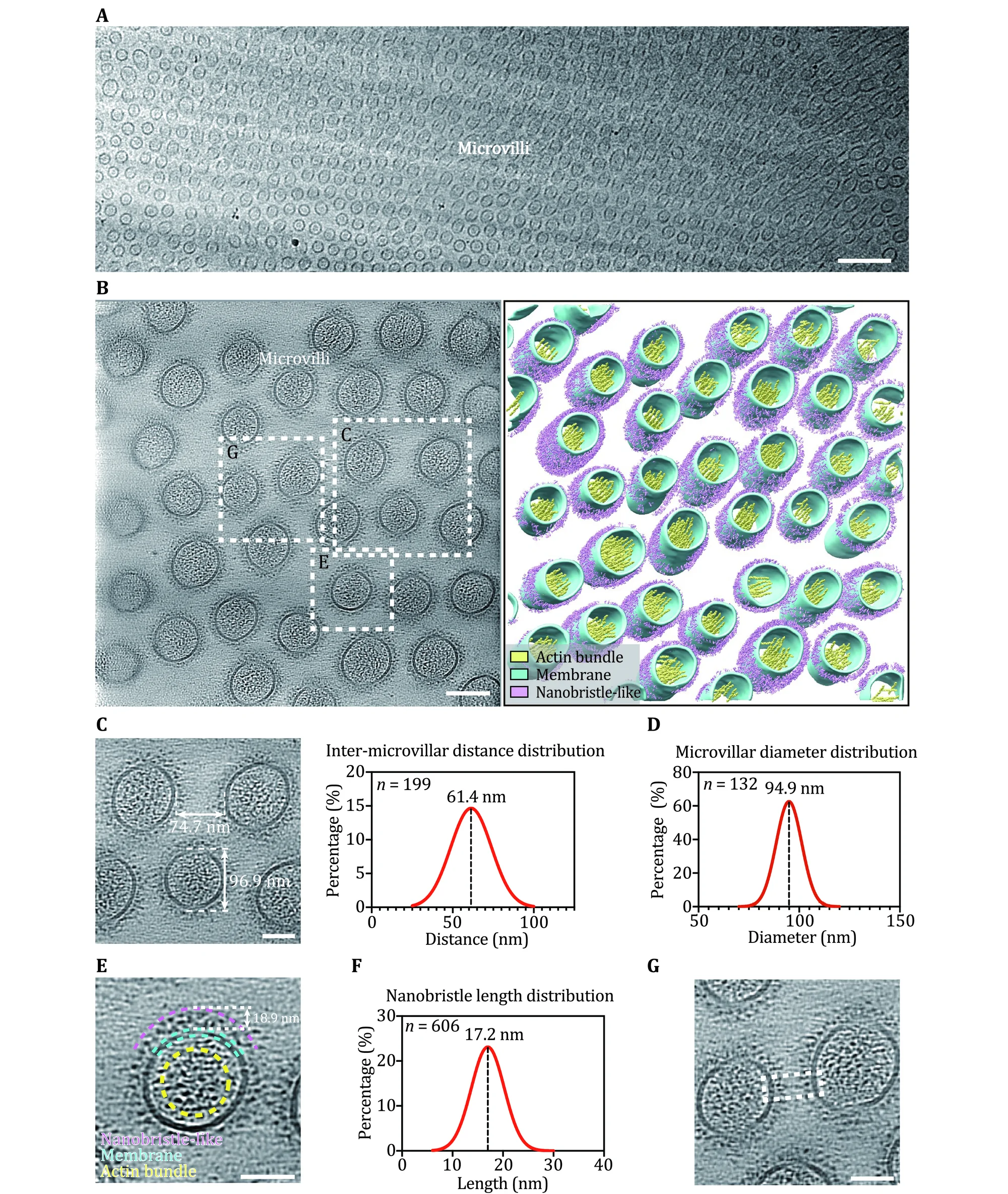
*In situ* cryo-ET analysis of microvilli on mouse intestinal epithelial cells. **A** TEM image of intestinal lamellae prepared with optimized cryo-FIB milling revealed the absence of non-vitreous ice formation and a uniform distribution of microvilli in the mouse intestine; scale bar = 100 nm. **B** Left: A representative tomographic slice of mouse intestinal microvilli, showing a uniform distribution with actin bundles in the core and protein densities on the surface. Right: 3D segmentation visualization of the microvillus membrane (blue), nanobristle-like structure (purple) and actin bundles (yellow); scale bar = 100 nm. **C**,**D** Microvilli exhibit a regular arrangement, evaluated by inter-microvillar distance (**C**) and microvillus diameter (**D**); scale bar = 50 nm. **E** An enlarged view of a representative microvillus from Panel B, showing nanobristle-like structures and the underlying actin bundle; the membrane and actin bundle are colored blue and yellow, respectively; scale bar = 50 nm. **F** Length distribution plot of nanobristle-like structures. **G** Longer nanobristle-like structures bridging adjacent microvilli; scale bar = 50 nm

We imaged the lamellae using a Titan Krios cryo-electron microscope and confirmed the vitrified state of the samples (Fig. 3A). From these preparations, 43 tomographic tilt series were collected from regions containing microvilli.

The reconstructed tomograms revealed microvilli organized in a quasi-regular lattice across the brush border (Fig. 3B). Quantitative analysis yielded an average inter-microvillar spacing of ~61.4 nm (Fig. 3C) and microvillar diameters ranging from 72.0 nm to 113.5 nm (Fig. 3D), values consistent with prior ultrastructural studies using conventional TEM (Beer *et al.* 2020; Revenu *et al.* 2012). Actin bundles within the microvillar core were clearly resolved (Fig. 3E), indicating well-preserved cytoskeletal architecture.

In addition to this canonical organization, each microvillus displayed lateral nanobristle-like projections. These structures exhibited morphological heterogeneity and frequent curvature, with lengths ranging from 9.2 to 34.7 nm (mean 17.2 nm) (Fig. 3F). In contrast to nanobristles in *C. elegans* (Zhu *et al.* 2022), the mammalian nanobristles displayed lower uniformity in density and rigidity and were approximately half the length of cadherin 8-based nanobristles in nematodes (~37.5 nm), suggesting differences in structural and mechanical properties. In rare cases, longer nanobristle-like filaments extended across the intermicrovillar space, connecting adjacent microvilli at distances of ~45.3 nm (Fig. 3G), closely matching the spacing of protocadherin-based intermicrovillar adhesion complexes in the mouse intestine (46.8 ± 8.9 nm) (Crawley *et al.* 2014). These features may facilitate intermicrovillar interactions or contribute to the stabilization of brush-border packing. While the precise molecular identity remains undetermined.

### Y-shaped microvilli

**Figure 4:**
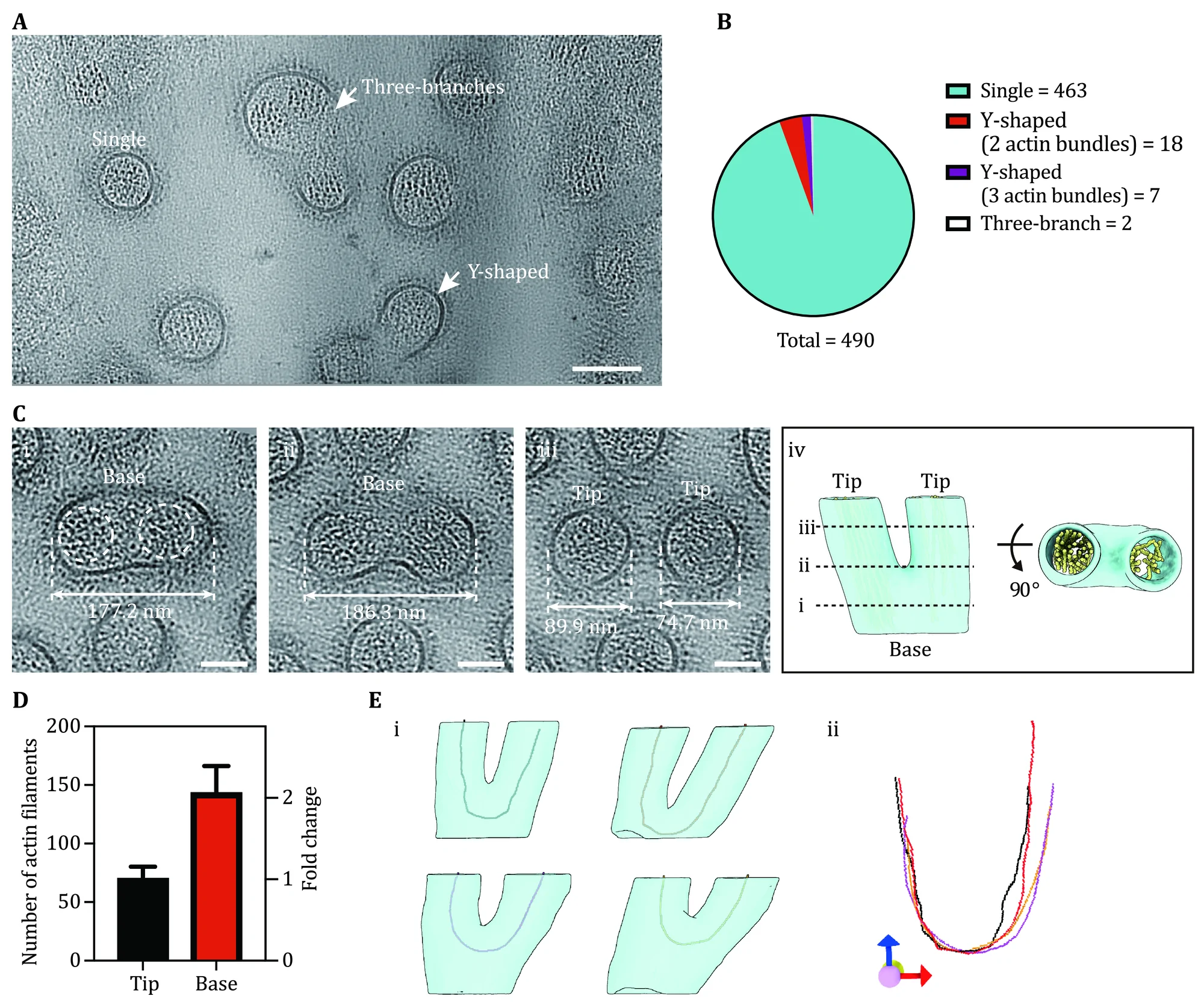
Analysis of branched microvilli. **A** A representative tomographic slice displaying three types of microvilli: single, Y-shaped, and three-branched microvilli; scale bar = 100 nm. **B** Quantitative distribution of different types of branched microvilli. **C** Sequential tomographic slices of a representative Y-shaped microvillus (i−iii) , and the corresponding 3D segmentation model (iv); scale bar = 50 nm. **D** Quantification of actin filaments at the two tips and the base of Y-shaped microvilli. **E** 3D illustration of branching angles in four representative Y-shaped microvilli

A major finding of this study is the identification of microvilli with branched morphologies. Among 490 microvilli analyzed from multiple tomograms, 27 exhibited branching (~5.5%), including both Y-shaped and three-branched configurations (Figs. 4A and 4B) (Zhu *et al.* 2022). This frequency is higher than that previously reported in *C. elegans* (~1.5%) and suggests that branching may occur more commonly in mammalian brush borders.

Sequential tomographic slices and 3D reconstructions revealed that Y-shaped microvilli consist of two distinct tips emerging from a single base (Fig. 4C). The base diameter was nearly twice that of a single tip, and actin filament bundles were arranged in separate clusters (Figs. 4C and 4D). Importantly, the number of filaments per daughter tip approximately matched the total number in the base, consistent with a tip-to-base division model in *C. elegans* (Zhu *et al.* 2022). This structural conservation supports the hypothesis that branched forms represent intermediates in microvillar renewal, rather than artifacts of sample preparation. Branching angles among Y-shaped microvilli were highly variable (Fig. 4E), suggesting flexibility in the geometry of division events.

### Diversity of branched microvilli beyond simple bifurcation

**Figure 5:**
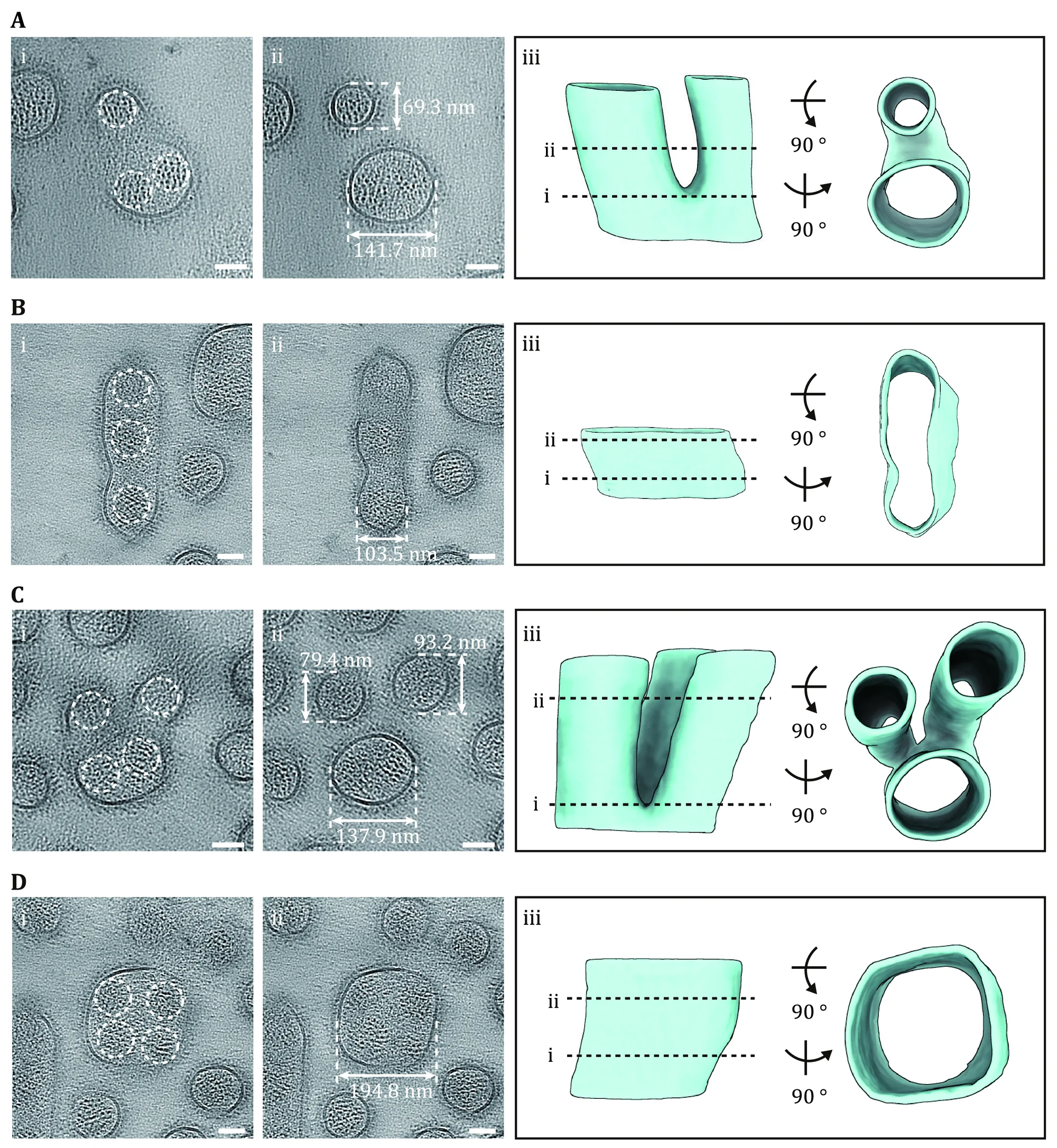
Diversity analysis of other types of branched microvilli. **A** Sequential tomographic slices (i−ii) and 3D segmentation (iii) of a typical Y-shaped microvillus revealed that one of the tips contains two actin clusters, along with three actin bundle clusters at the base. **B** Sequential tomographic slices (i−ii) and 3D segmentation (iii) of a typical Y-shaped microvillus showed three actin bundle clusters aligned linearly at the base. **C** Sequential tomographic slices (i−ii) and 3D segmentation (iii) of a typical three-branched microvillus revealed two actin clusters at one of the tips and four actin bundle clusters at the base. **D** Sequential tomographic slices (i−ii) and 3D segmentation (iii) of a typical three-branched microvillus showed four clusters of actin bundles at the base. Scale bars = 50 nm

In addition to canonical Y-shaped morphologies, we identified more complex branching states. Some Y-shaped microvilli contained three actin clusters at the base, with one daughter tip harboring two distinct filament bundles and appearing nearly twice the diameter of the other tip (Fig. 5A). In another case, three clusters were arranged linearly at the base, suggesting the potential to form three tips (Fig. 5B). Even more strikingly, we observed three-branched microvilli in which one tip contained two actin clusters (Fig. 5C), while the base harbored four distinct actin bundles (Fig. 5D). These morphologies indicate that a single microvillar base can give rise to multiple protrusions, potentially generating three or four new microvilli. Such diversity exceeds what has been described in *C. elegans* and in earlier electron microscopy studies, underscoring the higher morphological plasticity of mammalian brush borders.

Collectively, our findings point to a previously underappreciated level of structural heterogeneity in mammalian microvilli with an optimized cryo-ET workflow. Nanobristle-like projections may contribute to regulating intermicrovillar spacing and preventing excessive lateral fusion, analogous to their proposed role in *C. elegans*. Branched morphologies, in turn, provide direct structural evidence for a regenerative remodeling pathway in which new microvilli arise from the division of pre-existing ones. The combination of heterogeneous nanobristles and branching morphologies suggests a coordinated mechanism for maintaining brush-border density and functional capacity under conditions of epithelial turnover.

## DISCUSSION

Using an optimized tissue-level cryo-ET workflow, we captured intact brush-border architecture in the mouse small intestine and uncovered notable structural heterogeneity. High-quality tomograms resolved canonical actin-cored microvilli decorated by lateral nanobristle-like projections and a spectrum of branched morphologies − Y-shaped and more complex multi-branched forms. Of 490 microvilli surveyed, ~5.5% were branched, and the actin-bundle organization in these structures is consistent with a tip-to-base division model.

These observations have three immediate implications. First, the geometry and filament distribution of branched microvilli support a fission-like pathway for generating new protrusions, a mechanism that could enable rapid local increases in microvillus number without de novo nucleation of actin bundles. Second, the lateral nanobristle-like projections − although shorter and more heterogeneous than those described in *C. elegans* − may contribute to tip−tip interactions or spacing regulation, with occasional longer projections spanning intermicrovillar gaps at distances comparable to known adhesion complexes. Third, the variety of branching geometries (asymmetric daughters, linear multi-cluster bases, and three- or four-way branching) indicates that branching is a flexible, regulated process likely influenced by local mechanical and molecular cues.

The study has limitations. Cryo-ET provides static snapshots and cannot directly demonstrate the temporal progression of division events. Lamella thickness and sampling geometry may underrepresent or truncate branched structures, introducing sampling bias in frequency estimates. The heterogeneity and flexibility of nanobristles also precluded subtomogram averaging, leaving their molecular identity unresolved.

Addressing these limitations calls for complementary approaches: correlative live imaging followed by rapid cryo-immobilization to track dynamics; correlative volume EM or serial-section TEM with specific fluorescent labeling to capture continuous side views and improve frequency estimates; and targeted genetic or labeling experiments to test roles for protocadherins, tip-link components, and actin regulators. Larger tomogram datasets and strategies to enrich homogeneous nanobristle populations could enable molecular characterization by subtomogram averaging.

Methodologically, the HPF−cryo-CLEM−serial lift-out pipeline used here facilitated targeted sampling and reproducible lamella preparation while reducing operator dependency, and it should be broadly applicable to other tissues where *in situ* nanoscale architecture is of interest.

In summary, our *in situ* structural analyses reveal heterogeneous nanobristle projections and diverse branched microvillar morphologies in the mouse intestine, supporting a tip-to-base division mechanism and revealing significant brush-border plasticity. These findings motivate mechanistic and functional studies to define the molecular determinants and physiological contexts that govern microvillar branching.

## METHODS

### Intestine isolation and high-pressure freezing

The intestines were isolated from two-month-old CAG-RFP-EGFP-LC3 transgenic C57BL/6J mice (JAX stock #027139) as described by Weigmann *et al*. (Weigmann *et al.* 2007). A segment of approximately 1 mm × 1 mm was carefully excised using bone-cutting spring scissors (Fine Science Tools). The isolated intestinal segment was immediately transferred into a high-pressure freezing carrier with a 3-mm diameter and 300-μm deep cavity (Leica Microsystems). A drop of 40% Ficoll in PBS was added to the cavity to fill it completely, after which a sapphire disc was placed on top to seal the sample. The assembled sample sandwich was then promptly placed into the central well of the middle plate and transferred to the Leica EM ICE high-pressure freezer (Leica Microsystems) for rapid freezing. The frozen samples were subsequently stored in liquid nitrogen until further processing.

### Correlative light and electron microscopy

To precisely locate the microvilli in HPF samples embedded within the ice block in the carrier prior to cryo-FIB milling, cryo-CLEM was performed. The RFP and EGFP fluorescent signals from transgenic mice were utilized to identify the microvilli.

The frozen samples within the carrier were loaded into a plasma cryo-SEM/FIB system (Thermo Scientific Helios™ 5 Hydra CX DualBeam) equipped with an iFLM module and an EasyLift nanomanipulator. The isolated intestinal villi were imaged using the iFLM correlative imaging system at excitation wavelengths of 470 nm (GFP) and 565 nm (RFP), respectively. The fluorescent images were subsequently coregistered with the corresponding SEM and ion beam images using MAPS software (Thermo Scientific). Based on the identified location of the intestinal villi, the VOI was defined for subsequent trench milling procedures.

### Cryo-focused ion beam milling

In this study, we optimized a serial lift-out workflow requiring minimal user expertise; the detailed protocol was described in Tang *et al*. (Tang *et al*. 2025). The entire procedure can be divided into four main steps: sample block lift-out, receiver grid preparation, six-sided attachment, and fine milling.

#### Sample block lift-out

Prior to trench milling, a protective layer of metal-organic platinum was deposited on the sample surface using a gas injection system (GIS) to prevent damage from the high-energy ion beam. Trench milling was then performed to isolate all sides of the VOI, leaving only one side partially attached for controlled lift-out. Following the procedure described by Schiotz *et al*. (Schiøtz *et al.* 2024), a cross-section milling pattern was applied to the top, left, and bottom sides of the VOI, with dimensions of 50 µm × 23 µm × 10 µm (length × width × height), 5 µm × 50 µm × 10 µm, and 50 µm × 5 µm × 10 µm, respectively, based on the VOI dimensions (40 µm × 50 µm). These patterns were milled at a beam current of 60 nA using xenon plasma ions. Subsequently, the EasyLift nanomanipulator was inserted and attached to the top of the VOI via redeposition milling (Schreiber *et al.* 2018). The right side of the VOI was then milled with a similar cross-sectional pattern, fully detaching the sample block, which was then extracted by retracting the EasyLift needle.

#### Receiver grid preparation

A G100/400 rectangular mesh copper grid (Gilder Grids) was loaded on the left side of the shuttle, with the 100-mesh bars oriented horizontally. Three vertical 400-mesh bars were selected in the center region for milling. Patterns of 40 µm × 40−50 µm × 10 µm (length × width × z-depth), 40 µm × 8 µm × 10 µm, and 40 µm × 40−50 µm × 10 µm were applied, creating three empty regions; the pattern width was adjusted according to the sample block dimensions. The stage was tilted to seven degrees, and two cross-sectional milling patterns of 5 µm × 5 µm × 10 µm were applied to the central empty region from both sides, generating two plateaus for lamella attachment.

#### Six-sided attachment

The sample block was reinserted and positioned above the two plateaus. Initially, the block was attached to both sides via redeposition from the left and right sides of the two plateaus, followed by attachment from the bottom sides of the plateaus. A first lamella of approximately 4 µm thickness was then released from the sample block using a line milling pattern, and the remaining sample block was subsequently extracted. The lamella was further attached at the top left and right sides via vertical redeposition. This process was iterated until the entire volume of interest was sectioned. All redeposition patterns in this step were milled at a beam current of 4 nA.

#### Fine milling

The fine milling procedure was optimized based on Pöge *et al*. (Pöge *et al.* 2025). Initially, the front and back surfaces of the lamellae were milled away to expose a clean surface for subsequent processing. A second layer of metal-organic platinum was sputtered to provide additional protection. Fine milling was performed at a stage tilt of seven degrees. A notch milling pattern in the waffle method (Kelley *et al.* 2022) was first applied at 0.1 nA. This was followed by rough milling using rectangular patterns at 1 nA to a thickness of 2.5 µm, 0.3 nA to 1.5 µm, and 0.1 nA to 0.8 µm. Final polishing was performed at 30 pA, resulting in lamellae of approximately 200 nm in thickness.

### Cryo-ET data collection

Tomographic tilt series acquisition was performed using a Titan Krios G4 (Thermo Fisher Scientific) electron microscope operated at 300 kV, equipped with a Selectris Energy Filter and a Falcon 4i direct electron detector. During all acquisitions, an energy-selecting slit width of 20 eV was applied. Dose-symmetric tilt series were collected using an automated data collection strategy implemented with SerialEM (Hagen *et al.* 2017) and implementing PACE-tomo software (Eisenstein *et al.* 2023). The tilt series was acquired in movie mode using the .EER file format at a nominal magnification of 64,000x (corresponding to a pixel size of 2.33 Å) in counting mode. Tilting was performed from +60° to −60°, starting from a predetermined milling angle, with a tilt increment of 3°, resulting in a total dose of 120 e^−^/Å^2^. The target defocus range was set between −4 and −7 µm.

### Tomogram reconstruction and segmentation

Tilt series were processed using the Relion-5 pipeline (Burt *et al.* 2024). Motion correction was performed using the built-in algorithm implemented in Relion-5. CTF estimation was carried out with CTFFIND4 (Rohou and Grigorieff 2015). The CTF-corrected tilt series were aligned and reconstructed using the patch-tracking method in IMOD software (Mastronarde and Held 2017) at a binning factor of 6, resulting in a pixel size of 13.98 Å. Denoising was performed using cryo-CARE (Buchholz *et al.* 2019) for subsequent visualization and analysis.

Membrane segmentation was initially performed using Membrain-Seg (Lamm *et al.* 2025) and subsequently refined with Amira software (Thermo Fisher Scientific). Structure visualization and movie generation were conducted with UCSF ChimeraX (Pettersen *et al.* 2021).

### Quantification and statistical analysis

The length of the nanobristle-like structures decorating the microvilli was measured along their curved trajectories using the IMOD measurement module (Mastronarde and Held 2017). The distance between adjacent microvilli shown in the tomographic slices was determined with ImageJ software (Schneider *et al.* 2012). The statistical analysis of inter-microvillar distance and microvillar diameter was performed using PyCurv (Salfer *et al.* 2020) software, based on the 3D segmentations of the microvillar membrane obtained from Amira software (Thermo Fisher Scientific). Branching angles of Y-shaped microvilli were quantified by tracing the central axis of each branch within tomograms using the cylinder tracing tool in Amira. Coordinate data were subsequently processed with a custom MATLAB script to calculate angular relationships, and three-dimensional visualizations and plots were generated in ChimeraX. All plots and distribution analyses were generated using GraphPad Prism 10 (GraphPad Software, LLC).

### Data and materials availability

Representative tomograms will be available in the Electron Microscopy Data Bank.
